# Nanomaterials as Alternative Control Means Against Postharvest Diseases in Fruit Crops

**DOI:** 10.3390/nano9121752

**Published:** 2019-12-10

**Authors:** Sergio Ruffo Roberto, Khamis Youssef, Ayat Farghily Hashim, Antonio Ippolito

**Affiliations:** 1 Agricultural Research Center, Londrina State University, Londrina 86057-970, PR, Brazil; 2 Agricultural Research Center, Plant Pathology Research Institute, 9 Gamaa St, Giza 12619, Egypt; 3 Fats and Oils Department, National Research Centre, Cairo 12622, Egypt; aya_hashim43@yahoo.com.sg; 4 Department of Soil, Plant and Food Science, University of Bari “Aldo Moro”, 70121 Bari, Italy; antonio.ippolito@uniba.it

**Keywords:** nanomaterials, post-harvest diseases, nanotechnology, fruits

## Abstract

Post-harvest diseases of fruit and vegetables have to be controlled because of the high added value of commodities and the great economic loss related to spoilage. Synthetic fungicides are the first choice worldwide to control post-harvest diseases of fruit and vegetables. However, several problems and constraints related to their use have forced scientists to develop alternatives control means to prevent post-harvest diseases. Physical and biological means, resistance inducers, and GRAS (generally recognized as safe) compounds are the most important alternatives used during the last 20 years. Recently, nanomaterial treatments have demonstrated promising results and they are being investigated to reduce the utilization of synthetic fungicides to control post-harvest rot in fruit and vegetables. The collective information in this review article covers a wide range of nanomaterials used to control post-harvest decays related to each selected fruit crop including grape, citrus, banana, apple, mango, peach, and nectarine. Other examples also used are apricot, guava, avocado, papaya, dragon, pear, longan, loquat, jujubes, and pomegranate fruits.

## 1. Introduction

Post-harvest diseases of fruit and vegetables are commonly caused by several plant pathogenic microorganisms, including fungi and bacteria, leading to serious losses during storage and transportation. Nanotechnology, as a new approach, has been utilized in numerous applications in assorted fields of science including agriculture. The exceptional properties of nanomaterials make them a practical choice for sustainable horticulture in general and in particular post-harvest diseases of fruit and vegetable at the scientific level and industry stage [1].

### 1.1. Economic Importance of Post-Harvest Diseases

Generally, crop losses due to plant diseases are direct and indirect. Direct yield losses caused by pathogens and other agents such as insects, animals or weeds are responsible for crop losses varying between 20%–40% of worldwide agricultural production [2,3,4,5]. The aforementioned losses can be divided as follows: 18% losses come from animal pests; 16% due to microbial diseases in which 70%–80% of these losses are caused by plant pathogenic fungi; and 34% are due to weeds [5]. The economic losses due to the fungal diseases in the post-harvest chain are variable and not well documented, and they ranged from 30%–50% depending on the agricultural practices and location [6,7,8]. The Food and Agriculture Organization of the United Nations mentioned that 33% of the food delivered worldwide for human consumption is lost after harvest [9]. Those losses were distributed throughout the production network because pathogen-initiated diseases are the significant part of food wastage. 

### 1.2. Problems of Synthetic Fungicides

At present, the control of post-harvest decay of fruit and vegetables is achieved by the application of pre- and post-harvest chemical fungicides, such as imazalil, thiabendazole, pyrimethanil, and fludioxonil on citrus or boscalid and iprodione on grapes [10,11,12]. Chloride-based chemicals, when used as sanitation agents, can formulate chlorinated organic compounds including chloramines, dichloramines, and trichloromethanes, which are considered respiratory irritants supposed to be carcinogenic [13]. On the other hand, the broad pre- and post-harvest use of chemical fungicides has caused resistant strains of pathogens resulting in a breakdown of fungicide effectiveness [14,15]. In spite of the fact that these techniques are expensive and take time, they are important to extend the technical life of fungicides after some time [16,17]. Furthermore, consumers are worried about the utilization of chemical fungicides since their active ingredients and co-formulants have been related to health problems and ecological contamination [18]. 

### 1.3. Alternative Control Means and Their Mode of Action 

In the last two decades, numerous studies have been researching the impact of non-chemical applications, for example, irradiations, natural compounds, biocontrol agents, hot water/air application, and salts [9,10,14,19,20,21,22,23,24,25,26,27,28,29,30]. More details concerning the alternative control of post-harvest diseases were described including biological control (yeast antagonist, bacterial antagonist, botanicals), physical treatments (heat, UV-C, modified atmosphere, ozone treatment, electrolysed water), natural compounds (chitosan, oligochitosan, salts), essential oils (vervain oil, thyme oil, lemongrass oil, tea tree oil, oregano oil) [31,32].

The modes of action of those alternative control means against post-harvest diseases of fruit and vegetables have not been fully investigated. They can act directly on the pathogen, by inhibiting spore germination and mycelium growth, and altering morphological structures and reactive oxygen species (ROS) generation, spore mitochondrial membrane potential (MMP) and adenosine triphosphate (ATP) content. Understanding the mechanisms of action of those alternative control means is very important to help optimize their practical uses [33,34,35]. A detailed study had been carried out to investigate the mechanism of salts against gray mold on different hosts, showing that potassium bicarbonate, sodium silicate, and calcium chelate can change the morphology and function of mitochondria in the fungus. The aforementioned salts caused accumulation of ROS, reduced adenosine triphosphate content and decreased spore mitochondrial membrane potential, resulting in the loss of mitochondria role [36].

In addition to the direct mechanism, induction of resistance in the fruit and vegetable was also proposed. In nature, plants are frequently challenged by a diverse array of pathogenic microorganisms, but they are able to defend themselves against almost all of the attacks. In many cases, their protective mechanisms involve inducible defense reactions, such as the accumulation of hydrolytic enzymes, phytoalexins, or the production of structural barriers. The failure of plants to invoke such defense reactions may result in susceptibility to disease. Induced disease resistance has been defined as “the process of active resistance dependent on the host plant physical or chemical barriers, activated by biotic or abiotic agents” [37]. It is well documented that treating plants with various agents (e.g. virulent or avirulent pathogens, non-pathogens, cell wall fragments, plant extracts, and synthetic chemicals) can lead to the induction of resistance, both locally and systemically, to subsequent pathogen attacks [38]. Hence, there is a huge interest in exploring new strategies based on the activation of the plant defense mechanisms [39] as an alternative to traditional means for controlling diseases. 

The induction of plant defense reactions is presumed to be mediated by an initial natural recognition process between plants and pathogens, which involves the detection of certain structural features of incompatible pathogens by plant receptor-like molecules. This hypothesis is supported by the finding that certain compounds, known as elicitors, and isolated from pathogenic microorganisms, induce biochemical events characteristic of the resistance responses in plants [40].

### 1.4. Nanomaterials as Candidate to Reduce Fungicides Use

Nanomaterials have been proposed and had an accepting significance due to their particular properties and their critical applications in food and agriculture [41,42]. Advancements in nanotechnology have resulted from the effort of researchers and specialists working in isolated fields, for example, biological sciences, biotechnology, and chemistry. To overcome practical problems facing post-harvest diseases, nanobiology is vital and needed. Nanomaterials can be defined as wide range of materials that include specific substances, which have a length less than 100 nm at least [43,44] where a nanometer is one billionth of a meter. Different strategies can be utilized for preparation and synthesis of nanomaterials, however these techniques are extensively isolated into two fundamental classes including bottom-up and top-down methodologies. More details concerning nanomaterials categories (carbon-based, metal, ceramics, semiconductor, polymeric, lipid-based nanomaterials), synthesis, characterization of nanomaterials (morphological, structural, particle size and surface area, optical characterizations), properties and applications were described in [45]. In the field of post-harvest technology of fruits and vegetables, the nanotechnology approach is helpful for: controlling post-harvest diseases; introducing a new invention for packaging films; preventing impact of gases and unsafe rays; improving packaging appearance; and helping for labeling fresh products using the multiple chips (nanobiosensors). Nanotechnology was used to produce nanomaterials for use as antifungal agents in several commodities, including fruits and vegetables. Several nanomaterials have shown potential in post-harvest management and have been developed to control diseases in citrus, grapes banana, apple, mango, peach, and nectarine. 

## 2. Post-Harvest Diseases of Citrus 

World citrus production has reached 146,599,168 tons [46]. The main post-harvest diseases of citrus can be divided into two groups based on their initial infections: (i) diseases from field infection such as Alternaria rot, Brown rot, Phomopsis and Diplodia stem-end rot, and Anthracnose; and (ii) diseases due to post-harvest infection such as Penicillium decays, Aspergillus, Rhizopus, Sour rot, and Fusarium decays [47]. Green, blue, and whisker decays caused by *Penicillium digitatum* (Pers.:Fr.) Sacc., *P. italicum* Wehmer, and *P. ulaiense* (Hsieh, Su and Tzean), respectively, are the most important post-harvest diseases attacking citrus fruit worldwide [19,20,48]. The above post-harvest diseases are usually controlled by synthetic fungicide application in packinghouses. However, several constrains related to their use forced scientists to develop alternative means to control post-harvest diseases. The main problems are: (i) the development of pathogen resistance to fungicides; (ii) legislative restrictions on the use of fungicides; and (iii) public demand for fungicide free-products [12,32]. 

Clay-chitosan nanocomposite (CCNC) was prepared at different ratios of clay/chitosan and was tested against *P. digitatum* on ‘Valencia Late’ sweet orange (*Citrus sinensis* L. Osb.). In in vitro tests, a complete inhibition of the pathogen was achieved at 20 µg·mL^−1^ for clay/chitosan (1:0.5), clay/chitosan (1:1), and clay/chitosan (1:2). In in vivo trials, a complete inhibition of the disease was obtained, and a high reduction (around 70%) of lesion development was reported for clay/chitosan at 1:2 ratio at 20 µg·mL^−1^. Scanning electron microscopy showed that treatment with this nanocomposite caused severe collapse and irregular branching of hyphae in the apical part. In addition, CCNC caused a degradation of DNA of the tested pathogen at 20 µg·mL^−1^ [49].

In fact, increasing chitosan/silicate nanocomposites by inserting chitosan chains into interlayers of silicate can increase their mechanical properties. Polymer nanocomposites have gained significant interest due to their superior thermal and mechanical properties, as compared to polymer alone [50]. Polymer-clay nanocomposites are a category of hybrid materials composed of organic polymer matrices and nanoscale organophilic clay fillers [51]. Three kinds of composites named tactoids, intercalation, and exfoliation can be attained when nanoclay is integrated with a polymer [52]. Chitosan nanoparticles were considered to be effective elicitors of several host resistances to different plant pathogen fungi [53].

The impact of CCNC coating on quality parameters of lemons during cold storage was assessed [54]. Coated lemon fruits showed significant differences in the lowest value of total soluble solids and punch force. Also, the highest value of titratable acid, firmness and peel shear forces was observed. Coated lemon fruits had the lowest mass loss (5.3%), suggesting that the quality parameters of lemon fruits were enhanced by applying chitosan-clay nanocomposite throughout the cold-storage period. 

The quality of ‘Thomson’ navel oranges (*C. sinensis* L. Osb.) coated with CCNC edible coating and fogger-wax during storage was investigated [55]. The results of this research demonstrated that fruit coated with chitosan-clay had the highest pH, chroma, peel moisture, and firmness in comparison with the control. In addition, CCNC edible coating increased the resistance of oranges against fungal infections, and maintained the orange color and inner texture strength during the cold-storage period. 

When tangerine fruits were coated with chitosan or chitosan/montmorillonite, and stored at 10 °C for 11 days, chitosan coating inhibited the decay rate of fruits only at the first 5 days. However, when tangerine fruits were coated by chitosan/montmorillonite containing 1% (*w*/*w*), a lower decay percentage, lower mass loss, higher contents of total soluble solids and titratable acidity were recorded. At the end of the storage period, decay percentage of coated fruits was only 28.3%, which was 5.8% lower than control treatment suggesting that chitosan/montmorillonite coating can prolong the shelf life period of tangerine fruits [56]. In this research, the integration of montmorillonite was capable of enhancing the water vapor barrier parameters of chitosan coating and subsequently reduced mass loss of tangerine fruits.

Silver nanoparticles, with an average diameter of 10 ± 5 nm were tested in vitro against *Alternaria alternata*, *P. digitatum* and *Alternaria citri* isolated from citrus fruits [57]. This nanoparticle at 150 mg·L^−1^ showed a good antifungal efficacy against the three tested pathogenic fungi. The results of this study confirmed silver nanoparticles as promising nanomaterials, even if compared to iprodione or difenoconazole fungicides at the same concentration. To understand the mechanism in which silver nanoparticles works, it has been mentioned that it may cooperate with phosphorus and sulphur and their interaction may damage the DNA and proteins, and be consequential in cell death. 

In ‘Valencia Late’ sweet orange (*C. sinensis* L. Osb.), copper nanoparticles (48 nm) were tested against *P. digitatum* and *Fusarium solani.* The in vitro tests showed that a complete inhibition of *P. digitatum* was recorded at 20 µg·mL^−1^ for copper nanoparticles. The percentage of reduction in colony diameter of *P. digitatum* was 43%, 60%, and 100% for copper nanoparticles, respectively. A complete inhibition of *F. solani* was obtained at 60 µg·mL^−1^ copper nanoparticles. The in vivo experiments demonstrated that copper nanoparticles completely inhibited green mold, in a direct action. In addition, when copper nanoparticles and *P. digitatum* were added into separate wounds, the reduction was 88.5% for copper nanoparticles. For fusarium rot, in direct action, copper nanoparticles completely inhibited the rot development. The mechanism by which copper nanoparticles acted against the pathogen was explained by degradation of fungal DNA [58]. In fact, earlier investigations related to the mechanism of copper nanoparticles on plant pathogenic fungi have not been enough to explain the precise action. Indeed, it gives the impression to suppose that the small-size range of copper nanoparticles may possibly add to its antifungal effect, given that it can simply penetrate pathogen cell membrane and lead to cell death [59].

*Alternaria alternata* can attack a wide range of agricultural products causing black rot of mandarins and brown spot on citrus fruits. The ZnO nanoparticles (<50 nm) at concentrations from 0 mM to 15 mM were tested against a number of fungal contaminants, including *A. alternata* [60]. The pathogen was inhibited by ZnO nanoparticles at concentrations higher than 6 mM. In the presence of ZnO nanoparticles, clear morphological aberrations in the fungal structures were detected. 

On lemon fruits, TiO_2_ nanoparticles (7 nm) applied on polypropylene film were prepared to control *Penicillium expansum*. The fruits were enveloped in the prepared film and treated with irradiation by UVA during two-weeks storage period at 25 °C. The authors confirmed a noteworthy decrease of decay in lemons wrapped in prepared coated film as compared to the control. They also referred this efficacy to the accumulation of ROS from the UVA-irradiated TiO_2_ surfaces that suppress fungus development [61]. A summary of recent application of different nanomaterials against post-harvest disease of citrus is mentioned in Table 1.

## 3. Post-Harvest Diseases of Grapes

Table grape is susceptible to huge losses caused by *Botrytis cinerea*, the causal agent of gray mold. Fungal resistance has been repeatedly observed in fungus populations treated with fungicides to control this disease [62]. Several alternative control means have been proposed to be included for integrated pest management of post-harvest diseases including nanomaterials. 

For ‘Flame Seedless’ table grape (*Vitis vinifera* L.), [63] prepared and characterized five safe nanomaterials, including silica, chitosan, and copper nanoparticles (NPs) and their combination to be used against gray mold. The prepared nanomaterials have characteristics as follows: silica NPs of size ranged between 140 and 150 nm; silica/copper NPs of size ranged between 520–550 nm; copper NPs size range was 25–35 nm. Three concentrations for each nanomaterial were used in in vitro tests, with silica and chitosan NPs resulting the best. Under high inoculum density, silica, chitosan, copper, silica-copper and silica chitosan-copper NPs at concentration 3 g·L^−1^ were able to decrease the percentage of decayed berries by 56%, 50%, 8%, 57%, and 54%, respectively. However, under low inoculum pressure (natural infection) nanoparticles were adequate to decrease the percentage of decayed berries by 34%, 54%, 5%, 35% and 45%, respectively. The authors summarized that nanomaterials, i.e. silica and chitosan, are promising compounds for management of gray mold of table grape cv. Flame Seedless. The mechanism of chitosan and silica NPs was explained by asymmetrical branching of the pathogen hyphae in the apical section and the failure of linearity and irregular mycelia growth detected by scanning electron microscopy (SEM). Also, the two nanomaterials exhibited a genotoxicity supported by degradation of pathogen DNA. 

Later on, Youssef et al. conducted experiments against Botrytis mold on ‘Italia’ and ‘Benitaka’ table grapes (*V. vinifera* L.) with a novel formulation chitosan/silica nanocomposite [64]. The prepared nanomaterials had an average diameter of 200, 60 and 48 nm for chitosan nanoparticles, silica nanoparticles, and chitosan/silica nanocomposite, respectively. Combined applications of chitosan nanoparticles/silica nanoparticles showed a synergistic effect against gray mold. Under artificial conditions, chitosan/silica nanocomposite at 1% reduced the incidence of decay by 59% as compared to control. Moreover, when the formulated nanocomposite was applied in the field, as a preharvest application, it reduced Botrytis mold development by 59% and 83%, for ‘Italia’ and ‘Benitaka’ table grapes, respectively, giving results even better than the individual chitosan or silica nanoparticles alone. 

It is worth mentioning that most of researchers focused on the impact of treatments on various aspects, while the influence on fruit quality is ignored. For this reason, the authors studied the effect of this formulated nanocomposite on physicochemical properties of table grapes. The authors concluded that chitosan nanoparticles and chitosan/silica nanocomposite were able to preserve ‘Italia’ table grape bunches from mass loss, while no negative effect of those nanomaterials was observed in terms of fruit quality. To explain the mechanism of chitosan/silica nanocomposite several approaches were studied, including ROS, ATP content, and MMP of the pathogen spores. In particular, higher increase in fluorescent spores in presence of this formulated nanocomposite at 0.6% was detected as compared to the control. Also, it reduced the ATP content after 5, 15, and 30 min of incubation by 34%, 38%, and 35%, respectively as compared to control. In addition, MMP decreased, resulting in a loss of mitochondria function [64]. In addition, silica-silver nanosized (1–5 nm) was examined at 0.3–100 µg·mL^−1^ concentration against ten plant pathogenic fungi and bacteria, including *B. cinerea*, showing a complete inhibitory effect at 10 µg·mL^−1^ [65]. 

Recently, a coating based on pullulan and polymeric nanocapsules (153.9 nm) containing thyme (*Thymus vulgaris* L.) was prepared and applied to increase the shelf life of table grapes. Grapes coated with the formulated nanocapsules maintained their color, firmness, titratable acidity, and soluble solids content for longer as compared to untreated grapes. In grapes stored for 6 months, gray mold incidence was lower in coated fruits as compared to untreated ones. This formulated coating avoided the quick evaporation of volatile compounds and increased their persistence on the grape surfaces, thus delaying grape ripening and extending their shelf life [66]. 

*Aspergillus niger* is the most important fungus responsible for post-harvest diseases of many fresh fruits including apples, pears, peaches, citrus, grapes, figs, strawberries, mangoes, and melons [67]. The antifungal efficacy in SiO_2_/Ag_2_S nanocomposites (300 nm) was reported for the first time against *A. niger*. The results showed that SiO_2_/Ag_2_S nanocomposites suppress the fungal development and sporulation. The authors refereed this effect to the presence of Ag_2_S nanophases, and they observed a synergistic effect due to Ag_2_S antifungal centres and the SiO_2_ surfaces in supporting the adsorption of the fungus [68]. It has been recommended that for metallic Ag, there is interaction of silver species with the sulphydryl groups of respiratory enzymes in the plasma membranes of susceptible pathogens, causing changes in the membrane permeability [69]. SEM showed that *A. niger* hyphae become covered by SiO_2_/Ag_2_S nanomaterials when the fungus was exposed to the compound [68]. Another hypothesis to explain this mechanism was the binding of Ag^+^ to microbial genetic material [70].

In in vitro experiments, ZnO nanoparticles (70 ± 15 nm) were tested against *B. cinerea* at different concentrations (0, 3, 6 and 12 mmol·L^−1^). The growth of the pathogen was inhibited at concentrations greater than 3 mmol·L^−1^. The author hypothesized that ZnO nanoparticles affected cellular functions of *B. cinerea* causing deformation in pathogen hyphae [71].

In other in vitro trials, silver nanoparticles (38 nm), copper nanoparticles (20 nm), and silver-copper nanoparticles were examined at different concentrations (0, 1, 5, 10 and 15 mg·L^−1^) against *A. alternata* and *B. cinerea*. Fifteen mg·L^−1^ resulted in being the best concentration to inhibit both pathogens. Using SEM the authors demonstrated that the nanoparticles obviously damaged the hyphae and conidia of *A. alternata*. In *B. cinerea*, damage to the fungal hyphae surface was noted resulting in the discharge of internal cellular materials with shrinkage of pathogen hyphae. Regarding the cell wall components of *P. expansum*, a huge reduction in total sugar and protein was recorded while a little reduction in lipids was observed due to the application of silver nanoparticles. In case of *B. cinerea*, a highly decrease in total protein of culture filtrate and total protein and N-acetyl glucoseamine of cell wall was noted, while an increasing in total lipids for culture filtrate and cell wall was observed following silver nanoparticles treatments [72].

In another in vitro investigation, silver nanoparticles (50 nm) at different concentrations (25–100 mg·L^−1^) were prepared and combined with the fungicide tolclofosmethyl. This combination performed better than individual compounds against *B. cinerea*; in particular, the most significant inhibition for the pathogen was obtained when the ratio between silver nanoparticles to tolclofos-methyl was 100:10 [73]. 

Many scientists have investigated the development or preparation of nanomaterials coating formulations to extend post-harvest storage period. Regarding the packaging of red table grapes, a chitosan-TiO_2_ composite film (70 µm thickness,) was prepared and evaluated against *Escherichia coli, Staphylococcus aureus, Candida albicans,* and *A. niger*. The chitosan-TiO_2_ film (50–80 nm) was examined as packaging table grapes in order to avoid post-harvest infections and expand the shelf life period. In this study, table grape was preserved for 22 days with composite film compared to two weeks with bare chitosan film before mildew occurred [74]. Nanocomposites of chitosan/gelatin and silver nanoparticles (25–45 nm) were successfully prepared using the solution casting technique. The grapes wrapped with the nanocomposites hybrid film showed a fresh appearance without putridity, and the surface was kept smooth without any leakage of the grape juice. This nanocomposite led to extending the shelf life period for extra two weeks [75].

In in vitro trials, chitosan nanoparticles (128.3 nm size) were tested against five pathogenic bacteria, including *S. aureus, Listeria monocytogenes, Pseudomonas aeruginosa, Salmonella* spp., and *E. coli,* with minimum inhibition concentration ranging 2–3 g·L^−1^ [76]. Chitosan nanomaterials efficiently delayed the ripening process and led to reduced mass loss, soluble solids, and sugar contents and increased moisture retention and preservation of the titratable acidity values and sensory characteristics. Also, the lowest decay percentage was recorded in grapes stored under refrigeration as compared to grapes stored at room temperature, and the percentage was much higher than the decay rates found for grapes coated with chitosan nanoparticles. A summary of recent application of different nanomaterials against post-harvest disease of grapes is displayed in Table 2.

## 4. Post-Harvest Diseases of Banana 

Banana (*Musa acuminata* L.) is considered to be one of the most essential fruits grown in tropical and subtropical areas worldwide. As a climacteric fruit, it has a relatively short post-harvest period due to several processes that change quality loss after harvest [77]. Coating of banana fruit with chitosan combined with gum arabic at 1% delayed ripening by reducing the respiration rate and improved shelf life period for up to 33 days [78]. Banana peel extracts were considered as potential candidate for the synthesis of silver nanoparticles which had a good antibacterial activity against *E. coli, E. aerogenes, Klebsiella* sp. and *Shigella* sp. [79].

For banana fruits, chitosan nanoparticle (102.4–370 nm diameter) was synthesized, characterized and its coating effect on ripening process was investigated. The results of physical characteristics of bananas ripening demonstrated that banana coated with chitosan nanoparticles at concentration 0.2% had a slower skin discolouration by 2–3 days. Also, SEM showed a smoother skin formed on the coated banana when compared to untreated fruit [80]. In this study, it was mentioned that edible coating could avoid loss of humidity and aroma, inhibit the oxygen penetration into fruit tissues, and avoid the post-harvest pathogen growth. 

Banana anthracnose caused by *Colletotrichum musae* is the most important disease attacking banana fruits. The effectiveness of ajwain (*Trachyspermum ammi*) and neem (*Azadirachta indica*) leaf extracts of copper, silver, nickel and magnesium (68 nm) were tested in vitro against the causal agent of banana anthracnose. The results showed that ajwain-magnesium NPs, ajwain-nickel NPs at 0.2% and neem-silver NPs at 0.1% and 0.2% completely inhibited the spore germination of the pathogen. Silver-neem nanoparticles were examined in vivo against banana anthracnose disease at different concentrations, and spraying this nanomaterial at 0.2% showed the lowest anthracnose severity [81]. 

Chitosan NPs (121.2 nm) and chitosan at 1.25% can provide a good effect on post-harvest quality of banana (extend banana fruits up to several days), starch content, mass loss (delay ripening process), pulp to peel ratio, soluble solids, and sensory quality [82]. The mechanism of action using molecular analysis showed that 1-aminocyclopropane-1-carboxylate synthase (MaACS1) and 1-aminocyclopropane-1-carboxylate oxidase (MaACO) gene expression of coated bananas was lower than control treatment. This behavior was explained as ethylene production decreased because of the reduction of MA-ACS1 and MA-ACO expression; generally, ethylene regulates the expression of ripening related genes such as MA-ACS1 and MA-ACO [80]. 

Soybean protein isolate/cinnamaldehyde/ZnO nanocomposite was formulated using: soybean protein isolate, cinnamaldehyde, and ZnO NPs, which are well-known as inorganic nanomaterial with good mechanical properties, barrier capability, and great antimicrobial action. The soybean protein isolate nanocomposite film effectively delayed the ripening level and mass loss of banana fruits. The aforementioned nanocomposite coating delayed the damaging changes in fruit firmness, soluble solids, titratable acidity and sensory quality during storage period. The antifungal efficacy of formulated nanocomposite coating against *A. niger* was 1.25-fold higher than only protein isolate/cinnamaldehyde application, while the pure protein isolate solution alone had no inhibitory effect. This behavior indicated that the immediate use of cinnamaldehyde and ZnO nanoparticle produced a synergistic effect for banana coating application. The authors explained the mechanism of action as this formulated nanocomposite film triggered severe oxidative stress, leading to extreme accumulation of ROS in *A. niger* [83].

In a study performed by [84] in banana puree films, chitosan nanoparticles (88.79 nm diameter) were added to pectin and glycerol as plasticizer. The results showed that pectin and glycerol had an important role in promoting extension and film handability. The incorporation of nanoparticles promoted noticeable improvement of the mechanical properties and acted in reducing the water vapor permeation rate by 21% for films processed with pectin and up to 38% for films processed without pectin, as compared to the control. A summary of recent application of different nanomaterials against post-harvest disease of banana is mentioned in Table 3.

## 5. Post-Harvest Diseases of Apple 

’Golab Kohanz’ (*Malus domestica* Bork.) is a well-known sensitive and highly perishable apple with a restricted shelf life storage. Chitosan nanoemulsion (≤100 nm diameter) was used at 0.2 and 0.5% concentrations as coating on apple fruit stored at 1 ± 1 °C and 85%–90% relative humidity for 9 weeks. Coating with chitosan nanoemulsion significantly reduced mass loss, respiration level, ethylene rate and peroxidase activity as compared to control. Also, chitosan nanoemulsion prolonged the quality and avoided mass loss of apple during storage. In particular, mass loss of control fruit was 5.5% while it was 3.4% and 2.2% for coated fruit with 0.2% and 0.5% nanochitosan, respectively [85].

In fresh-cut ‘Gala’ apple (*M. domestica* Bork.), different sizes of chitosan-based nanoparticles (110 and 300 nm) were examined in terms of microbiological profile, color, polyphenol oxidase and peroxidase, and firmness. Apple coated with 110 nm-chitosan NPs demonstrated higher antimicrobial action and reduced total microbial contamination levels of a wide range of pathogens evaluated as log colony forming units (CFU)·g^−1^, as compared to other treatments. Apple browning slices coated with normal chitosan or uncoated-control was slightly higher than slices coated with nanochitosan [86].

A novel nanopackaging was developed by [87] by coating polyvinyl chloride film with ZnO NPs (200–400 nm diameter) and its effect on fresh-cut ‘Fuji’ apple (*M. domestica* Bork.) preservation quality was tested. Fresh-cut apple stored with ZnO NPs packaging had lower fruit decay percentage (21.5%) as compared to control (42.4%). Also, nanopackaging reduced the accumulation of malondialdehyde and decreased polyphenoloxidase and pyrogallol peroxidase activities as compared to control fruit. The primary appearance of apple slices was maintained and the browning index was avoided in nanopackaging ‘Fuji’ apple fruits stored at 4 °C for 12 days. The nanopackaging was also able to maintain the level of soluble solids and titratable acidity. 

Regarding fresh-cut apple, a poly-lactic acid film incorporated with ZnO nanoparticle was prepared as a novel nanopackaging film and tested on the quality attributes of apple [88]. The nanofilm had a higher water vapor permeability and lower oxygen permeability as compared to the pure poly-lactic acid film. Although nanofilms had the highest mass loss at the end of storage compared to the pure poly-lactic acid film, it provided a better preservation of color, firmness, total phenolic content and sensory quality. Concerning the microbial profile (CFU·mL^−1^), the combination of ZnO NPs and poly-lactic acid was able to inhibit the growth of pathogens including bacteria, molds and yeasts in fresh-cut apple stored at 4 °C for two weeks. 

For ’Red Delicious’ apple (*M. domestica* Bork.), nano-calcium and calcium chloride were applied at different concentrations in the field five times, starting 70 days following full-bloom untill one month before apple harvesting. Firmness, titratable acidity, total phenolic content, total antioxidant activity, and fiber content increased in apple fruits treated with nano-calcium and calcium chloride as compared to untreated fruit. The quality of apples treated with nano-calcium was better than apple fruits treated with calcium chloride in all investigated parameters during the storage period. The authors proved that nano-calcium decreased cell wall enzyme activities including polygalacturonase, pectin methylesterase and β-galactosidase [89]. 

ZnO NPs (70 ± 15 nm) were tested against *P. expansum*, the causal agent of post-harvest blue mold of apple, at different concentrations (0, 3, 6 and 12 mmol·L^−1^). The pathogen growth was inhibited at concentrations higher than 3 mmol·L^−1^. The mechanism of ZnO NPs on the pathogen proved to be related to the prevention of growth of conidiophores and conidia and death of hyphae [71]. The antifungal activity of ZnO NPs (sizes < 50 nm) against *P. expansum* was also investigated by [90]. The results showed that the minimum inhibitory concentration and non-inhibitory concentration were 9.8 and 1.8 mM, respectively. 

Fresh-cut ‘Red Delicious’ apples were coated with tocopherol/nopal mucilage (*Opuntia ficus indica*) nanoemulsion (less 1000 nm) and had a significant impact on pectin methylesterase and polyphenol oxidase activity in the fresh-cut apples. The authors confirmed that nopal mucilage performed as an encapsulant that improves the activity of α-tocopherol. Therefore, color, firmness and mass loss were maintained during refrigerated storage [91]. A summary of recent application of different nanomaterials against post-harvest disease of apple was mentioned in Table 4.

## 6. Peach and Nectarine

Concerning ‘Shan-i-Punjab’ peach (*Prunus persica* L.), chitosan-rice-starch nanocomposite films was fabricated by integration of synthesized silver and ZnO NPs via a solvent-casting method [90]. The aforementioned nanocomposite was evaluated in vitro against *E. coli* and *S. aureus* bacteria. The nanocomposite films incorporated with NPs reduced peach-surface microbial density and improved the shelf life of packed peach fruit as compared to unpackaged or packaged control. In particular, the microbial count on the peach surface was high for the control treatment and lowest for peach fruit packaged with commercial silver NPs. In the packinghouse, chitosan–rice starch nanocomposite films were able to maintain peach quality properties. The antimicrobial performance of formulated nanocomposites against peach microorganism seemed to be bactericidal and bacteriostatic [92].

In ‘Red Top’ and ‘Anjiry’ peaches (*P. persica* L.), and ‘Red Gold’, ‘Songlu’ and ‘Independence’ nectarines (*P. persica* L.), potassium permanganate coated with nanozeolites (nano-absorbent granules) was prepared to check its role on quality properties and shelf life after removing ethylene from the storage environment. Treatment with zeolite nanoparticles coated with potassium permanganate in a machine circulating the ethylene-containing air, avoided mass loss, reduced the firmness and contamination, and increased shelf life and the quality of peach and nectarine samples during storage [93].

Fruits of ‘Desert Red’ peach (*P. persica* L.) were coated with chitosan NPs (50 nm) at 0.2, 0.4, and 0.8% and directly stored at 0 ± 1 °C and 90–95% RH for 28 days. The results demonstrated that chitosan NPs at 0.4% gave the lowest percentage of fruit decay and soluble solids/titratable acidity ratio compared with other treatments. Also, when chitosan nanoparticle was used at 0.8%, fruit mass loss was reduced and fruit pulp firmness was maintained [94]. A summary of recent application of different nanomaterials against post-harvest disease of peach and nectarine is shown in Table 5.

## 7. Mango

Colletotrichum is a huge genus covering a wide range of significant species (*Colletotrichum gloeosporioides* (Penz.) Penz. & Sacc) causing post-harvest anthracnose diseases in various horticultural crops, including mango (*Mangifera indica* L.). Colletotrichum was designated as the 8th most important phytopathogenic fungus worldwide based on supposed scientific and economic importance [95]. Chitosan-silver NPs composite (495–616 nm diameter) was prepared and the silver NPs were distributed in the composite with size 10–15 nm. The composite exhibited higher antifungal effect against the conidial germination of *C. gloeosporioides*. In particular, a complete inhibition of spore germination was obtained when chitosan-silver NPs composite was applied at 100 µg·mL^−1^. Moreover, on detached mango fruit cv. Alphonso, in vivo experiments indicated that anthracnose was significantly suppressed by the aforementioned composite. A 45.7% and 71.3% reduction of anthracnose disease was achieved by chitosan-silver nanoparticle composite at 0.5% and 1%, respectively [96]. 

Recently, Dubey et al. (2019) developed a nanocomposite edible film containing *Aloe vera* gel, glycerol and ZnO NPs solutions [97]. Mango fruits were coated and stored for nine days at room temperature and quality parameters were evaluated. The overall results showed that increasing the concentration of ZnO NPs led to a reduction of mass loss (%), increasing to titratable acidity, increasing of ascorbic acid content in fruit, maintaining a low soluble solids content, decreasing the pH, increasing thickness and increasing the transmittance and elongation (%) of the formulated films. 

Copper oxychloride-conjugated silver NPs were synthesized using neem extract and tested for their activity against the causal agent of mango anthracnose (*C. gloeosporioides*) by [98]. The resulted nonmaterial had spherical shape and an average particle size of 21–25 nm. The nanomaterials displayed the highest growth inhibition of the pathogen (~187%) as compared to copper oxychloride. The obtained results confirmed that this nanomaterial can be successfully used as alternative control means for controlling anthracnose disease in mango. A summary of recent applications of different nanomaterials against post-harvest disease of mango is shown in Table 6. 

## 8. Apricot, Guava, Avocado, Papaya and Dragon Fruits 

In apricot fruits (*Prunus armeniaca* L.), firmness is considered to be one of the most important factors for consumers. Application of calcium at 1% delayed the changes of physicochemical properties and the depolymerization of chelate-soluble pectin through storage period [99]. Ethylene is considered to be the most important issue in ripening and its production and buildup during storage is capable to cause severe damage. NPs confirmed that potassium permanganate and zeolite based nano-molecular filters were able to increase apricot shelf life period and improve marketing value [100].

On guava fruits (*Psidium guava* L.), Garcia-Betanzos et al. (2018) developed a solid lipid nanoparticle/xanthan gum coating (276 nm diameter). ‘Media China’ guava fruits were immersed in the formulated coating and then stored at 10 °C and 85% RH up to 32 days [101]. The results showed that guavas treated with solid lipid NPs at 65 g·L^−1^ showed the lowest value (234 mg/100 g of fruit) of ascorbic acid loss (34%). In the same context, guava fruits were coated with a film-forming dispersion (245 nm diameter) containing 3 g·L^−1^ of xanthan gum, 5 g·L^−1^ of glycerol and solid lipid NPs at 5 or 10 g·L^−1^ stored at 7 °C and 85% of RH for 22 days. The results revealed that solid lipid NPs at 5 g·L^−1^ showed the best performance in terms of fruit quality. In particular, coatings decreased the browning index and firmness loss of guava fruits, and subsequently extended fruit shelf-life period [102,103]. 

Recently, microencapsulated starch containing ascorbic acid was used to coat guava fruits by dipping in two concentrations of microcapsules of 6.25% or 12.5%, and then storing at 4 °C and 65% RH for 12 days. The authors proved that fruit coated with the control solution and solutions containing 6.25% and 12.5% microcapsules showed significant changes in the content of soluble solids, pH, and titratable acidity. However, significant decreases in respiration rate, firmness and mass were observed in fruit coated with 12.5%, 6.25% and control fruit, respectively. Coated guavas demonstrated a decrease in ripening and reduced changes in fruit color as compared to control [104].

Some metal nanomaterials such as MgO and ZnO composites (52–219 nm) were tested against *C. gloeosporioides*, the causal agent of anthracnose on papaya and avocado tropical fruits. All nanomaterials significantly inhibited the germination of conidia and the action proved to be fungicidal since the minimum inhibitory concentrations were equal to the minimum fungicidal concentration. Regarding the radial growth, *C. gloeosporioides* from papaya was more resistant to benomyl than *C. gloeosporioides* from avocado, and had minimum inhibitory concentrations of 0.625 and 0.312 μg·mL^−1^, respectively. The authors summarized that the tested nanomaterials can be used as alternatives to control anthracnose [105].

Nanoemulsions (NE) are prepared using aqueous phase, oils, surfactants, and co-surfactants [106]. It was demonstrated that chitosan in the form of nanoemulsions exhibited better antifungal efficacy when compared to its raw form. The effect of chitosan in the form of nanoemulsions (200–1000 nm) against *C. musae* and *C. gloeosporioides*, the causal agents of anthracnose of tropical fruits including banana, papaya and dragon fruits was investigated. The fruits treated with conventional chitosan showed higher disease incidence and severity when compared to fruits treated with chitosan nanoemulsions. Nevertheless, in banana fruits, the start of anthracnose symptoms was delayed and the symptoms appeared only after two weeks of cold storage period. While in papaya and dragon fruits, the symptoms appeared after seven days of cold storage. Also, it is worth mentioning that chitosan-loaded nanoemulsion treatments did not demonstrate any negative effects on the quality of tested fruits [107].

In the effect of carnauba (*Copernicia prunifera*) wax nanoemulsion (42 nm diameter) was examined on physicochemical properties of ‘Golden’ papayas (*Carica papaya* L.). When the fruits were coated with carnauba wax nanoemulsion at 2.4% concentration and stored for 9 days at 22 ± 1 °C and 60%–70% RH, the mass loss and decay incidence were reduced as compared to control treatment [108].

In dragon fruits (*Hylocereus polyrhizus*), chitosan with various droplet sizes (200 to 1000 nm) at 1.0% was tested against *C. gloeosporioides* in term of conidia growth, mycelia dry weight and sporulation. The obtained results showed that chitosan (600 nm diameter) performed the best in inhibiting spore germination (91.5%), decreasing dry mycelia mass (0.47 g) and sporulation (7.5%) when compared locally chitosan with inhibition in spore germination by 67.9%, mycelia dry mass, 0.76 g and sporulation, 25.4%. The findings obtained suggested that chitosan loaded nanoemulsions can act as a promising nanomaterial for controlling anthracnose disease on dragon fruits [109]. A summary of recent application of different nanomaterials against post-harvest disease of apricot, guava, avocado, papaya and dragon fruits is given in Table 7.

## 9. Pear, Longan, Loquat, Jujube and Pomegranate Fruits 

In ‘D’Anjou’ and ‘Bartlett’ pears (*Pyrus communis* L.), cellulose nanocrystal reinforced 2% chitosan coatings were tested to delay the ripening and quality deterioration of post-harvest green pear during three weeks at room temperature or five months of cold storage, respectively. At room temperature, the cellulose nanocrystal-chitosan coating at 5% significantly delayed green chlorophyll degradation of pear peels, prevented internal browning, reduced senescence scalding, and improved fruit firmness. During the cold storage period, coating showed a viable effect on delaying fruit post-harvest quality deterioration compared to a commercial control. The coating robustly remained on the surface of the pear fruit and offered a better gas barrier as compared to other coatings [110].

Concerning longan fruits (*Dimocarpus longan* Lour.), chitosan/nano-silica coating at 1% was tested on conservation quality ’Shijia’ fruits. The film formulated was able to prolong fruit shelf life, decreasing browning index and delay mass loss (by 38%–60%). The nanomaterials were capable of inhibiting the increase of malondialdehyde quantity and decrease polyphenoloxidase and peroxidase enzymatic activity in fresh fruit. When fruits were coated with chitosan/nano-silica film, an increase of the contents of soluble solids and titratable acidity, and the rate of decrease in ascorbic acid was much lower in treated fruit as compared to control [111]. 

A porous chitosan/nano-silica film was used to coat ‘Baiyu’ loquat fruits (*Eriobotrya japonica* Lindl.), which were stored at 5 °C for 40 days. The nanomaterial formulated was able to delay the internal browning and mass loss of treated loquat fruit as compared to control. When fruits were coated with chitosan/nano-silica film, glucose (11.31 to 12.76 g·kg^−1^) and fructose (33.20 to 44.37 g·kg^−1^) contents increased. The mechanism of chitosan/nano-silica film was explained by the low levels of malondialdehyde and membrane permeability detected in coated fruits and also by the lower activity of enzymatic profile such as phenylalanine ammonia-lyase, polyphenoloxidase and lipoxidase [112].

Regarding ‘Dongzao’ jujube fruits (*Ziziphus jujuba* Mill.), chitosan film (1%) combined with nano-silicon dioxide (0.04%) was examined on the qualitative parameters of fruits. Jujube fruits were coated and stored for 32 days at room temperature. The red indices, mold incidence, mass loss (36.4% lower), and respiration level decreased as compared to control. In particular, decay incidence decreased by 46.2% as compared to untreated treatment. Jujube fruits coated with chitosan film combined with nano-silicon dioxide showed lower phenylalnine ammonialyase activity (PAL) and superior activities of superoxide dismutase, peroxidase, and catalase [113].

Concerning ‘Ardestani’ pomegranate fruits (*Punica granatum*), nano-zinc chelate and nano- boron chelate (23–80 nm diameter) were prepared at different concentrations and applied in the field before full bloom. Both nanomaterials improved pomegranate tree nutrient status. When a low amount of those nanomaterials (34 mg nano-boron chelate/tree or 636 mg nano-zinc chelate/tree) was applied in the field, an increase in fruit yield was observed, and subsequently higher number of fruit per tree was obtained [114]. A summary of recent application of different nanomaterials against post-harvest disease of pear, longan, loquat, jujube and pomegranate fruits is mentioned in Table 8.

**Figure 1:**
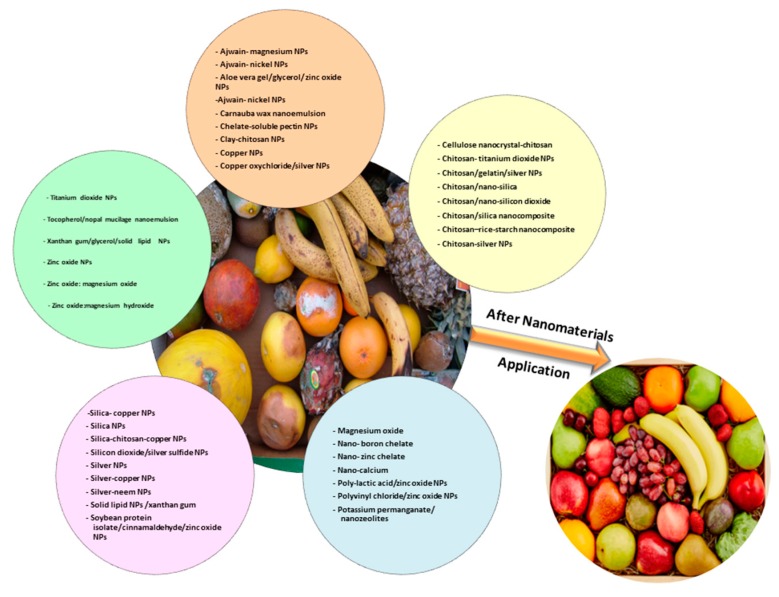
Different nanomaterials used against post-harvest fruit diseases.

Nanomaterials used against post-harvest diseases of fruit mentioned on this review are summarized in Figure 1.

## 10. Conclusions and Future Prospects

There are several and easy post-harvest practices such as suitable harvest timing, fruit washing and disinfection, categorization, wrapping, pre-cooling and suitable transportation and distribution, etc. to reduce post-harvest losses. However, those practices are not always efficient at maintaining fruit quality after harvest. Fungicide application is still one of the most important means to control post-harvest disease of fresh fruits to extend their shelf-life. One of the most important factors for successful management of post-harvest diseases is the synthesis of low-cost nanomaterials with high efficiency for better storage conditions with maintaining the fruit quality. For this reason, a lot of effort is needed to enhance using nanomaterials as alternative control means for post-harvest disease management. Nanomaterials have been exploited as the novel generation of a revolution in the field of post-harvest technology in general and particularly disease management. A wise application of nanomaterials is needed since their risks and hazards are still unknown and not fully studied. Future investigations should look at possible human health effects since some materials, such as TiO_2_, have been considered to produce colon cancer. The size of the nanomaterials, as an important characteristic, was missing in some studies. Accordingly, we suggest that researchers provide the size in the future studies to enable other scientists to properly compare the nanoparticles between themselves and then plan the most efficient new design. Several factors including fruit species, dose and kind of nanomaterials, treatment timing, fruit-ripening stage and others can affect the efficacy of nanomaterials. Finally, the targeting groups of this review article are under- and post-graduate students, researchers, farmers, packing line and/or storage operators and all who are interested in post-harvest pathology/nanomaterial interaction.

## Figures and Tables

**Table 1:** Recent application of different nanomaterials used as alternative control means to manage post-harvest disease of citrus.

Fruit/Cultivar	Scientific Name	Nanomaterial	Objective	Size (nm)	Ref.
Valencia Late orange	*Citrus sinensis* L. Osb.	Clay-chitosan nanocomposite	Antifungal	NM *	[49]
Lemon	*Citrus limon* L. Osb.	Chitosan-clay nanocomposite	Coating	NM *	[54]
Thomson navel orange	*C. sinensis* L. Osb.	Chitosan-clay nanocomposite	Edible coating	NM *	[55]
Tangerine	*Citrus tangerine* Hort. ex Tanaka	Chitosan/montmorillonite	Coating/antifungal	NM *	[56]
Washington navel orange	*C. sinensis* L. Osb.	Silver nanoparticles	Antifungal	10 ± 5	[57]
Valencia Late	*C. sinensis* L. Osb.	Copper nanoparticles	Antifungal	48	[58]
-	*C. sinensis* L. Osb.	ZnO nanoparticles	Antifungal	<50	[60]
Lemon	*C. limon* L. Osb.	TiO_2_ nanoparticles	Coating/antifungal	7	[61]

NM * = size of the nanomaterials was not mentioned.

**Table 2:** Recent application of different nanomaterials used as alternative control means to manage post-harvest disease of grapes.

Cultivar/Type	Scientific Name	Nanomaterials	Objective	Size (nm)	Ref.
Flame Seedless table grape	*Vitis vinifera* L.	Silica nanoparticles (NPs), Copper NPs, Silica-copper NPs, Chitosan NPs	Antifungal	Si (140–150) Cu (25–35) Si-Cu (520–550)	[63]
Italia and Benitaka table grapes	*V. vinifera* L.	Chitosan/silica nanocomposite	Antifungal	48	[64]
Grapes	*V. vinifera* L.	Nanocapsules/*Thymus vulgaris* L.	Coating/antifungal	153.9	[66]
-	*V. vinifera* L.	SiO_2_/Ag_2_S nanocomposites	Antifungal	300	[68]
-	*V. vinifera* L.	ZnO-nanoparticles	Antifungal	70 ± 15	[71]
-	*V. vinifera* L.	Silver-nanoparticles, copper-nanoparticles and silver-copper-nanoparticles	Antifungal	Silver (38) Copper (20)	[72]
-	*V. vinifera* L.	Silver-nanoparticles	Antifungal	50	[73]
Red grapes	*V. vinifera* L.	Chitosan-TiO_2_ composite	Antimicrobial	50–80	[74]
Red grapes	*V. vinifera* L.	Chitosan/gelatin and silver nanoparticles	Coating	25–45	[75]
Grapes	*Vitis labrusca* L.	Chitosan nanoparticles	Edible coating	128.3	[76]

**Table 3:** Recent application of different nanomaterials used as alternative control means to manage post-harvest disease of banana.

Group/Cultivar	Scientific Name	Nanomaterials	Objective	Size (nm)	Ref.
-	*Musa acuminata* L.	Chitosan/gum arabic	Coating	NM *	[78]
Cavendish bananas AAA group	*M. acuminata* L.	Chitosan-nanoparticles	Edible coating	102.4–370	[80]
-	*M. acuminata* L.	Ajwain-magnesium nanoparticles, Ajwain-nickel nanoparticles and Silver-neem nanoparticles	Antifungal	68	[81]
*M. acuminata* AAA group	*M. acuminata* L.	Chitosan-nanoparticles	Edible coating	121.2	[82]
-	*M. acuminata* L.	Soybean protein isolate/cinnamaldehyde/ZnO nanoparticles	Antifungal/film coating	NM *	[83]
Nanica	*Musa cavendishii* Lamb.	Chitosan-nanoparticles	Coating film	88.79	[84]

NM * = size of the nanomaterials was not mentioned.

**Table 4:** Recent application of different nanomaterials used as alternative control means to manage post-harvest disease of apple.

Cultivar	Scientific Name	Nanomaterials	Objective	Size (nm)	Ref.
Golab Kohanz	*Malus domestica* Bork.	Chitosan-nanoemulsion	Coating	≤100	[85]
Gala	*M. domestica* Bork.	Chitosan-nanoparticles	Coatings/antimicrobial	110–300	[86]
Fuji	*M domestica* Bork.	Polyvinyl chloride/ZnO nanoparticle	Nanopackaging coating	200–400	[87]
Yunnan ZhaoTong	*M. domestica* Bork.	Poly-lactic acid/ZnO nanoparticle	Nanopackaging coating	NM *	[88]
Red Delicious	*M. domestica* Bork.	Nano-calcium	Coating	NM *	[89]
-	*M. domestica* Bork.	ZnO nanoparticles	Antifungal	70 ± 15	[71]
-	*M. domestica* Bork.	ZnO nanoparticles	Antifungal	<50	[90]
Red Delicious	*M. domestica* Bork.	Tocopherol/nopal mucilage nanoemulsion	Encapsulant	<1000	[91]

NM * = size of the nanomaterials was not mentioned.

**Table 5:** Recent application of different nanomaterials used as alternative control means to manage post-harvest disease of peach and nectarine.

Cultivar	Scientific Name	Nanomaterials	Objective	Size (nm)	Ref.
Shan-i-Punjab peach	*Prunus persica* L.	Chitosan–rice-starch nanocomposite	Antimicrobial	NM *	[92]
Red Top and Anjiry peaches	*P. persica* L.	Potassium permanganate coated with nanozeolites	Removal of the ethylene	NM *	[93]
Red Gold, Songlu and Independence nectarines	*P. persica* var. *nucipersica* L.	Potassium permanganate coated with nanozeolites	Removal of the ethylene	NM *	[93]
Desert Red peach	*P. persica* L.	Chitosan nanoparticles	Antifungal	50	[94]

NM * = size of the nanomaterials was not mentioned.

**Table 6:** Recent application of different nanomaterials used as alternative control means to manage post-harvest disease of mango.

Cultivar	Scientific Name	Nanomaterials	Objective	Size (nm)	Ref.
Alphonso	*Magnifera indica* L.	Chitosan-silver nanoparticle composite	Antifungal	495–616	[96]
Dasheri	*M. indica* L.	Aloe vera gel, glycerol and ZnO nanoparticles	Film coating	NM *	[97]
-	*M. indica* L.	Copper oxychloride-conjugated AgNPs	Antifungal	21–25	[98]

NM * = size of the nanomaterials was not mentioned.

**Table 7:** Recent application of different nanomaterials used as alternative control means to manage post-harvest disease of apricot, guava, avocado, papaya and dragon fruits.

Fruit	Scientific Name	Cultivar	Nanomaterials	Objective	Size (nm)	Ref.
Apricot	*Prunus armeniaca* L.	Jinhong	Chelate-soluble pectin nanostructural	Coating	NM *	[99]
Apricot	*P armeniaca* L.	-	Potassium permanganate coated with nanozeolites	Extend shelf life	NM *	[100]
Guava	*Psidium guajava* L.	Media China	Solid lipid nanoparticles/xanthan gum	Coating	276	[101]
Guava	*P. guajava* L.	Media China	Xanthan gum, glycerol and solid lipid nanoparticles	Coating	245	[102]
Guava	*P. guajava* L.	-	Microencapsulated starch/ascorbic acid	Coating	NM *	[104]
Avocado	*Persea americana*	in vitro	ZnO, MgO and ZnO: MgO and ZnO:Mg(OH)_2_	Antifungal	52–219	[105]
	*Carica papaya*					
Banana	*Musa acuminata* L.	Pisang Berangan	Chitosan-loaded nanoemulsions	Antifungal	200–1000	[107]
Papaya	*C. papaya* L.	AAA Group Solo				
Dragon	*Hylocereus polyrhizus*	Red				
		Jaina				
Papaya	*C. papaya* L.	Golden	Carnauba wax nanoemulsions	Coating	42	[108]
Dragon	*H. polyrhizus*	in vitro	Chitosan-loaded nanoemulsions	Antifungal	200–1000	[109]

NM * = size of the nanomaterials was not mentioned.

**Table 8:** Recent application of different nanomaterials used as alternative control means to manage post-harvest disease of pear, longan, loquat, jujube and pomegranate fruits.

Fruit	Scientific Name	Cultivar	Nanomaterials	Objective	Size (nm)	Ref.
Pear	*Pyrus communis* L.	D’Anjou and Bartlett	Cellulose nanocrystal-chitosan	Coating	NM *	[110]
Longan	*Dimocarpus longan* Lour.	Shijia	Chitosan/nano-silica	Coating	NM *	[111]
Loquat	*Eriobotrya japonica* Lindl.	Baiyu	Chitosan/nano-silica	Coating	NM *	[112]
Jujubes	*Ziziphus jujuba* Mill.	Dongzao	Chitosan film/nano-silicon dioxide	Coating	NM *	[113]
Pomegranate	*Punica granatum*	Ardestani	Nano-zinc chelate and nano-boron chelate	Nutrients	23–80	[114]

NM * = size of the nanomaterials was not mentioned.

## References

[1] Feregrino-Perez A.A., Magaña-López E., Guzmán C., Esquivel K. (2018). A general overview of the benefits and possible negative effects of the nanotechnology in horticulture. Sci. Hortic..

[2] Teng P.S., Krupa S.V. Assessment of losses which constrain production and crop improvement in agriculture and forestry. Proceedings of the E. C. Stackman Commemorative Symposium.

[3] Teng P.S., Teng P.S. (1987). Crop Loss Assessment and Pest Management.

[4] Oerke E.C., Dehne H.W., Schönbeck F., Weber A. (1994). Crop Production and Crop Protection: Estimated Losses in Major Food and Cash Crops.

[5] Oerke E.C. (2006). Crop losses to pests. J. Agric. Sci..

[6] Ladaniya M.S. (2008). Citrus Fruit.

[7] Youssef K., Roberto S.R. (2014). Applications of salt solutions before and after harvest affect the quality and incidence of post-harvest gray mold of ‘Italia’ table grapes. Post-Harvest Biol. Technol..

[8] Youssef K., Roberto S.R. (2014). Salt strategies to control Botrytis mold of’ Benitaka’ table grapes and to maintain fruit quality during storage. Post-Harvest Biol. Technol..

[9] Gastavsson J., Cederberg C., Sonesson U. (2011). Global Food Losses and Food Waste.

[10] Stammler G., Brix H.D., Nave B., Gold R., Schoefl U., Dehne H.W., Deising H.B., Gisi U., Kuck K.H., Russell P.E., Lyr H. (2008). Studies on the biological performance of boscalid and its mode of action. Proceedings of the Modern Fungicides and Antifungal Compounds V.

[11] Romanazzi G., Feliziani E. (2014). Botrytis cinerea (Gray Mold). Post-Harvest Decay.

[12] Palou L. (2018). Post-harvest treatments with GRAS salts to control fresh fruit decay. Horticulturae.

[13] Fallanaj F., Sanzani S.M., Zavanella C., Ip polito A. (2013). Salt addition improves the control of citrus post-harvest diseases using electrolysis with conductive diamond electrodes. J. Plant Pathol..

[14] Hao W., Li H., Hu M., Yang L., Rizwan-ul-Haq M. (2011). Integrated control of citrus green and blue mold and sour rot by *Bacillus amyloliquefaciens* in combination with tea saponin. Post-Harvest Biol. Technol..

[15] Sánchez-Torres P., Tuset J.J. (2011). Molecular insights into fungicide resistance in sensitive and resistant *Penicillium digitatum* strains infecting citrus. Post-Harvest Biol. Technol..

[16] Vitale A., Panebianco A., Polizzi G. (2016). Baseline sensitivity and efficacy of fluopyram against Botrytis cinerea from table grape in Italy. Ann. Appl. Biol..

[17] Piccirillo G., Carrieri R., Polizzi G., Azzaro A., Lahoz E., Fernández-Ortuño D., Vitale A. (2018). in vitro and in vivo activity of QoI fungicides against *Colletotrichum gloeosporioides* causing fruit anthracnose in Citrus sinensis. Sci Hortic..

[18] Nicolopoulou-Stamati P., Maipas S., Kotampasi C., Stamatis P., Hens L. (2016). Chemical Pesticides and Human Health: The Urgent Need for a New Concept in Agriculture. Front. Public Health.

[19] Youssef K., Ligorio A., Nigro F., Ippolito A. (2012). Activity of salts incorporated in wax in controlling post-harvest diseases of citrus fruit. Post-Harvest Biol. Technol..

[20] Youssef K., Ligorio A., Sanzani S.M., Nigro F., Ippolito A. (2012). Control of storage diseases of citrus by pre- and post-harvest application of salts. Post-Harvest Biol. Technol..

[21] Youssef K., Sanzani S.M., Myrta A., Ippolito A. (2014). Effect of a novel potassium bicarbonate-based formulation against Penicillium decay of oranges. J. Plant Pathol..

[22] Talibi I., Boubaker H., Boudyach E.H., Ait Ben Aoumar A. (2014). Alternative methods for the control of post-harvest citrus diseases. J. Appl. Microbiol..

[23] Lachhab N., Sanzani S.M., Fallanaj F., Youssef K., Nigro F., Boselli M., Ippolito A. (2015). Protein hydrolysates as resistance inducers for controlling green mould of citrus fruit. Acta Hortic..

[24] Fallanaj F., Sanzani S.M., Youssef K., Zavanella C., Salerno M.G., Ippolito A. (2015). A new perspective in controlling post-harvest citrus rots: The use of electrolyzed water. Acta Hortic..

[25] Salem E.A., Youssef K., Sanzani S.M. (2016). Evaluation of alternative means to control post-harvest Rhizopus rot of peaches. Sci. Hortic..

[26] Jeong R.D., Chu E.H., Lee G.W., Cho C., Park H.J. (2016). Inhibitory effect of gamma irradiation and its application for control of post-harvest green mold decay of Satsuma mandarins. Int. J. Food Microbiol..

[27] Hussien A., Ahmed Y., Al-Essawy A.H., Youssef K. (2018). Evaluation of different salt amended electrolysed water to control post-harvest moulds of citrus. Trop. Plant Pathol..

[28] Hussien A., Al-Essawy A., Abo Rehab M., Youssef K. (2017). Preliminary investigation of alkaline and acidic electrolysed water to control Penicillium species of Citrus. Citrus Res. Technol..

[29] Youssef K., Roberto S.R., Colombo R.C., Canteri M.G., Abd-Elsalam K.A. (2019). Acibenzolar-S-methyl against Botrytis mold on table grapes in vitro and in vivo. Agronomy Sci. Biotechnol..

[30] Youssef K., Hussien A. (2020). Electrolysed water and salt solutions can reduce green and blue molds while maintain the quality properties of ‘Valencia’ late oranges. Post-Harvest Biol. Technol..

[31] Wisniewski M., Droby S., Norelli J., Liu J., Schena L. (2016). Alternative management technologies for post-harvest disease control: The journey from simplicity to complexity. Post-Harvest Biol. Technol..

[32] Sanzani S.M., Ippolito A., Xu X.M., Fountain M. (2019). New techniques for managing post-harvest diseases of fruit. Integrated Management of Diseases and Insect Pests of Tree Fruit.

[33] Droby S., Wisniewski M., Macarisin D., Wilson C. (2009). Twenty years of post-harvest biocontrol research: Is it time for a new paradigm?. Post-Harvest Biol. Technol..

[34] Youssef K., Sanzani S.M., Ligorio A., Ippolito A., Terry L.A. (2014). Sodium carbonate and bicarbonate treatments induce resistance to post-harvest green mould on citrus fruit. Post-Harvest Biol. Technol..

[35] Youssef K., Sanzani S.M., Ligorio A., Fallanaj F., Nigro F., Ippolito A. (2015). Biochemical and transcriptomic changes associated with induced resistance in citrus fruits treated with sodium salts. Acta Hortic..

[36] Youssef K., Roberto S.R., de Oliveira A.G. (2019). Ultra-structural alterations in Botrytis cinerea−the causal agent of gray mold−treated with salt solutions. Biomolecules.

[37] Kloepper J., Tuzun S., Kuć J. (1992). Proposed definitions related to induced disease resistance. Biocontrol Sci. Technol..

[38] Walters D.R., Newton A.C., Lyon G.D. (2005). Induced resistance: Helping plants to help themselves. Biologist.

[39] Sharma H.C., Crouch J.H., Sharma K.K., Seetharama N., Hash C.T. (2002). Applications of biotechnology for crop improvement: Prospects and constraints. Plant Sci..

[40] Yoshikawa M., Yamaoka N., Takeuchi Y. (1993). Elicitors: Their Significance and Primary Modes of Action in the Induction of plant defence reactions. Plant Cell Physiol..

[41] Alghuthaymi M.A., Ali A.A., Hashim A.F., Abd-Elsalam K.A. (2016). A Rapid Method for the Detection of *Ralstonia solanacearum* by Isolation DNA from Infested Potato Tubers Based on Magnetic Nanotools. Philipp. Agric. Sci..

[42] Sharma S., Jaiswal S., Duffy B., Jaiswal A.K. (2019). Nanostructured Materials for Food Applications: Spectroscopy, Microscopy and Physical Properties. Bioengineering.

[43] Laurent S., Forge D., Port M., Roch A., Robic C., Vander Elst L., Muller. R.N. (2008). Magnetic iron oxide nanoparticles: Synthesis, stabilization, vectorization, physicochemical characterizations, and biological applications. Chem. Rev..

[44] Sekhon B.S. (2014). Nanotechnology in agri-food production: An overview. Nanotechnol. Sci. Appl..

[45] Khan I., Saeed K., Khan I. (2017). Nanoparticles: Properties, applications and toxicities. Arab. J. Chem..

[46] (2019). FAOSTAT. http://www.fao.org/faostat/en/#data/QC.

[47] Ippolito A., Nigro F., De Cicco V., Bertolini P., Salerno M.G. (2009). Agrumi (pp. 181–195). Postharvest Pathology of Plant Products.

[48] Youssef K., Ahmed Y., Ligorio A., D’Onghia A.M., Nigro F., Ippolito A. (2010). First report of *Penicillium ulaiense* as a post-harvest pathogen of orange fruit in Egypt. Plant Pathol..

[49] Youssef K., Hashim A.F. (2020). Inhibitory Effect of Clay/Chitosan Nanocomposite against *Penicillium digitatum* on Citrus and its Possible Mode of Action. Jordan J. Biolog. Sci..

[50] Kumar S., Jog J.P., Natarajan U. (2003). Preparation and characterization of poly(methyl methacrylate)–clay nanocomposites via melt intercalation: The effect of organoclay on the structure and thermal properties. J. Appl. Polym. Sci..

[51] Kim K.Y., Lim H.J., Park S.M., Lee S.J. (2003). Synthesis and characterization of high impact polystyrene/organically modified layered silicate nanocomposites. Polymer Korea.

[52] Xu Y., Ren X., Hanna M.A. (2006). Chitosan/Clay Nanocomposite Film Preparation and Characterization. J. Appl. Polym. Sci..

[53] Pichyangkura R., Chatchawan S. (2015). Bio stimulant activity of chitosan in horticulture. Sci. Hortic..

[54] Taghinezhad E., Ebadollahi A. (2017). Potential application of chitosan-clay coating on some quality properties of lemon during storage. Agric. Eng. Int..

[55] Khoshtaghaza M.H., Taghinezhad E. (2017). Investigation effect of particle Nano coating on storage quality properties of Thomson orange. J. Food Sci. Technol. Mys..

[56] Xu D., Qin H., Ren D. (2018). Prolonged preservation of tangerine fruits using chitosan/montmorillonite composite coating. Post-Harvest Biol. Technol..

[57] Abdelmalek G.A.M., Salaheldin T.A. (2016). Silver Nanoparticles as a Potent Fungicide for Citrus Phytopathogenic Fungi. J. Nanomed. Res..

[58] Youssef K., Hashim A.F., Margarita R., Alghuthaymi M.A., Abd-Elsalam K.A. (2017). Antifungal Efficacy of Chemically-Produced Copper Nanoparticles Against *Penicillium digitatum* and *Fusarium solani* on Citrus Fruit. Philip. Agric. Sci..

[59] Ingle A.P., Duran N., Rai M. (2013). Bioactivity, mechanism of action, and cytotoxicity of copperbased nanoparticles: A review. Appl. Microbiol. Biotechnol..

[60] Sardella D., Gatt R., Valdramidis V.P. (2017). Physiological effects and mode of action of ZnO nanoparticles against post-harvest fungal contaminants. Food Res. Int..

[61] Maneerat C., Hayata Y. (2006). Antifungal activity of TiO2 photocatalysis against *Penicillium expansum in vitro* and in fruit tests. Int. J. Microbiol..

[62] Jacometti M.A., Wratten S.D., Walter M. (2010). Review: Alternatives to synthetic fungicides for *Botrytis cinerea* management in vineyards. Aust. J. Grape Wine Res..

[63] Hashim A.F., Youssef K., Abd-Elsalam K.A. (2019). Ecofriendly nanomaterials for controlling gray mold of table grapes and maintaining post-harvest quality. Eur. J. Plant Pathol..

[64] Youssef K., de Oliveira A.G., Tischer C.A., Hussain I., Roberto S.R. (2019). Synergistic effect of a novel chitosan/silica nanocomposites-based formulation against gray mold of table grapes and its possible mode of action. Int. J. Biol. Macromol..

[65] Park H.J., Kim S.H., Kim H.J., Choi S.H. (2006). A new composition of nanosized silica–silver for control of various plant diseases. Plant Pathol. J..

[66] Piña-Barrera A.M., Álvarez-Román R., Báez-González J.G., Amaya-Guerra C.A., Rivas-Morales C., Gallardo-Rivera C.T., Galindo-Rodríguez S.A. (2019). Application of a multisystem coating based on polymeric nanocapsules containing essential oil of *Thymus vulgaris* L. to increase the shelf life of table grapes (Vitis vinifera L.). IEEE Trans. Nanobiosci..

[67] Plascencia-Jatomea M., Yépiz-Gómez M.S., Velez-Haro J.M. (2014). Aspergillus spp. (Black Mold). Post-Harvest Decay, Control Strategies.

[68] Fateixa S., Neves M.C., Almeida A., Oliveira J., Trindade T. (2009). Anti-fungal activity of SiO_2_/Ag_2_S nanocomposites against *Aspergillus niger*. Colloids Surf. B. Biointerfaces..

[69] Semeykina A.L., Skulachev V.P. (1990). Submicromolar Ag^+^ increases passive Na^+^ permeability and inhibits the respiration-supported formation of Na^+^ gradient in Bacillus FTU vesicles. FEBS Lett..

[70] Keleher J., Bashant J., Heldt N., Johnson L., Li Y. (2002). Photo-catalytic preparation of silver-coated TiO_2_ particles for antibacterial applications. World J. Microbiol. Biotechnol..

[71] He L., Liu Y., Mustapha A., Lin M. (2011). Antifungal activity of zinc oxide nanoparticles against *Botrytis cinerea* and *Penicillium expansum*. Microbiol. Res..

[72] Ouda S.M. (2014). Antifungal Activity of Silver and Copper Nanoparticles on Two Plant Pathogens, *Alternaria alternata* and *Botrytis cinerea*. Res. J. Microbiol..

[73] Derbalah A.S., Elkot G.A., Hamza A.M. (2012). Laboratory evaluation of botanical extracts, microbial culture filtrates and silver nanoparticles against *Botrytis cinerea*. Ann. Microbiol..

[74] Zhang X., Xiao G., Wang Y., Zhao Y., Su H., Tan T. (2017). Preparation of chitosan-TiO_2_ composite film with efficient antimicrobial activities under visible light for food packaging applications. Carbohydr. Polym..

[75] Kumar S., Shukla A., Baul P.P., Mitra A., Halder D. (2018). Biodegradable hybrid nanocomposites of chitosan/gelatin and silver nanoparticles for active food packaging applications. Food Packag. Shelf. Life.

[76] Melo C.B.N.F., de MendonçaSoares B.L., Diniz K.M., Leal C.F., Canto D., Flores M.A.P., da Costa Tavares-Filho H.J., Galembeck A., Stamford M.T.L., Stamford-Arnaud T.M. (2018). Effects of fungal chitosan nanoparticles as eco-friendly edible coatings on the quality of post-harvest table grapes. Post-Harvest Biol. Technol..

[77] Zhang H., Yang S., Joyce D.C., Jiang Y., Qu H., Duan X. (2010). Physiology and quality response of harvested banana fruit to cold shock. Post-Harvest Biol. Technol..

[78] Maqbool M., Ali A., Alderson P.G., Zahid N., Siddiqui Y. (2011). Effect of a Novel Edible Composite Coating Based on Gum Arabic and Chitosan on Biochemical and Physiological Responses of Banana Fruits during Cold Storage. J. Agric. Food Chem..

[79] Bankar A., Joshi B., Kumar A.R., Zinjarde S. (2010). Banana peel extract mediated novel route for the synthesis of silver nanoparticles. Colloids Surf. A Physicochem. Eng. Asp..

[80] Esyanti R.R., Zaskia H., Amalia A., Nugrahapraja d.H. (2019). Chitosan Nanoparticle-based coating as post-harvest technology in banana. J. Phys. Conf. Ser..

[81] Jagana D., Hegde Y.R., Lella R. (2017). Green nanoparticles: A novel approach for the management of banana anthracnose caused by *Colletotrichum musae*. Int. J. Curr. Microbiol. Appl. Sci..

[82] Lustriane C., Dwivany F.M., Suendo V., Reza M. (2018). Effect of chitosan and chitosan-nanoparticles on postharvest quality of banana fruits. J. Plant Biotechnol..

[83] Li J., Sun Q., Sun Y., Chen B., Wu X., Le T. (2019). Improvement of banana post-harvest quality using a novel soybean protein isolate/cinnamaldehyde/zinc oxide bionanocomposite coating strategy. Sci. Hortic..

[84] Martelli M.R., Barros T.T., de Moura M.R., Mattoso L.H., Assis O.B. (2013). Effect of chitosan nanoparticles and pectin content on mechanical properties and water vapor permeability of banana puree films. J. Food Sci..

[85] Gardesh A.S.K., Badii F., Hashemi M., Ardakani A.Y., Maftoonazad N., Gorji A.M. (2016). Effect of nanochitosan based coating on climacteric behavior and post-harvest shelf-life extension of apple cv. Golab Kohanz. LWT-Food Sci. Technol..

[86] Pilon L., Spricigo P.C., Miranda M., de Moura M.R., Assis O.B.G., Mattoso L.H.C., Ferreira M.D. (2015). Chitosan nanoparticle coatings reduce microbial growth on fresh-cut apples while not affecting quality attributes. Int. J. Food Sci. Technol..

[87] Li X., Li W., Jiang Y., Ding Y., Yun J., Yao T., Zhang P. (2011). Effect of nano-ZnO-coated active packaging on quality of fresh-cut ‘Fuji’ apple. Int. J. Food Sci. Technol..

[88] Li W., Li L., Cao Y., Lan T., Chen H., Qin Y. (2017). Effects of PLA Film Incorporated with ZnO Nanoparticle on the Quality Attributes of Fresh-Cut Apple. Nanomaterials.

[89] Ranjbar S., Rahemi M., Ramezanian A. (2018). Comparison of nano-calcium and calcium chloride spray on post-harvest quality and cell wall enzymes activity in apple cv. Red Delicious. Sci. Hortic..

[90] Sardella D., Gatt R., Valdramidis V.P. (2018). Assessing the efficacy of zinc oxide nanoparticles against *Penicillium expansum* by automated turbidimetric analysis. Mycology.

[91] Zambrano-Zaragoza M.L., Gutiérrez-Cortez E., Del Real A., González-Reza R.M., Galindo-Pérez M.J., Quintanar-Guerrero D. (2014). Fresh-cut red delicious apples coating using tocopherol/mucilage nanoemulsion: Effect of coating on polyphenol oxidase and pectin methylesterase activities. Food Res. Int..

[92] Kaur M., Kalia A., Thakur A. (2017). Effect of Biodegradable Chitosan-rice-starch Nanocomposite Films on Post-Harvest Quality of Stored Peach Fruit. Starch.

[93] Masoumeh E., Behzad G., Yousef R.K., Mehrnaz E., Naser B. (2015). Effect of the Potassium Permanganate Coated Zeolite Nanoparticles on the Quality Characteristic and Shelf Life of Peach and Nectarine. J. Agric. Technol..

[94] Gad M.M., Zagzog O.A., Hemeda O.M. (2016). Development of Nano-Chitosan Edible Coating for Peach Fruits *Cv.* Desert Red. Int. J. Environ..

[95] Dean R., Van Kan J.A., Pretorius Z.A., Hammond-Kosack K.E., Di Pietro A., Spanu P.D., Rudd J.J., Dickman M., Kahmann R., Ellis J. (2012). The top 10 fungal pathogens in molecular plant pathology. Mol. Plant Pathol..

[96] Chowdappa P., Gowda S., Chethana C.S., Madhura S. (2014). Antifungal activity of chitosan-silver nanoparticle composite against *Colletotrichum gloeosporoides* associated with mango anthracnose. Afr. J. Microbiol. Res..

[97] Dubey P.K., Shukla R.N., Srivastava G., Mishra A.A., Pandey A. (2019). Study on Quality Parameters and Storage Stability of Mango Coated with Developed Nanocomposite Edible Film. Int. J. Curr. Microbiol. App. Sci..

[98] Raghavendra S.N., Raghu H.S., Divyashree K., Rajeshwara A.N. (2019). Antifungal efficiency of copper oxychloride-conjugated silver nanoparticles against *Colletotrichum gloeosporioides* which causes anthracnose disease. Asian J. Pharm. Clin. Res..

[99] Liu H., Chen F., Yang H., Yao Y., Gong X., Xin Y., Ding C. (2009). Effect of calcium treatment on nanostructure of chelate-soluble pectin and physicochemical and textural properties of apricot fruits. Food Res. Int..

[100] Emadpour M., Kalaj Y.R. (2009). Effect of ethylene absorption using nano-particles on the storage and quality characteristics of apricot. Agron. Hortic..

[101] Garcia-Betanzos C.I., Hernández-Sánchez H., Quintanar-Guerrero D., Galindo-Pérez M.J., Zambrano-Zaragoza M.L. (2018). Influence of solid lipid nanoparticle/xanthan gum coatings on compositional and enzymatic changes in guava (*Psidium guajava* L.) during ripening. Acta. Hortic..

[102] Zambrano-Zaragoza M.L., Mercado-Silva E., Ramirez-Zamorano P., Cornejo-Villegas M., Gutiérrez-Cortez E., Quintanar-Guerrero D. (2013). Use of solid lipid nanoparticles (SLNs) in edible coatings to increase guava (*Psidium guajava* L.) shelf-life. Food Res. Int..

[103] González-Reza R.M., Pérez-Olivier M.S., Miranda-Linares V., Zambrano-Zaragoza M.L. (2018). Effect of solid lipid nanoparticles coating on shelf life of refrigerated fresh-cut guava. Acta. Hortic..

[104] Martinez-Ortiz M.A., Palma-Rodriguez H.M., Montalvo-Gonzalez E., Sayago-Ayerdi S.G., Utrilla-Coello R., Vargas-Torres A. (2019). Effect of using microencapsulated ascorbic acid in coatings based on resistant starch chayotextle on the quality of guava fruit. Sci. Hortic..

[105] De la Rosa-García S.C., Martínez-Torres P., Gómez-Cornelio S., Corral-Aguado M.A., Quintana P., Gómez-Ortíz N.M. (2018). Antifungal Activity of ZnO and MgO Nanomaterials and Their Mixtures against *Colletotrichum gloeosporioides* Strains from Tropical Fruit. J. Nanomater..

[106] Adnan A., Mohammad R., Farhan J.A., Zeenat I., Roop K.K., Aqil M., Sushama T. (2009). Nanoemulsion components screening and selection: A technical note. AAPS Pharm. Sci. Tech..

[107] Zahid N., Ali A., Manickam S., Siddiqui Y., Maqboo M. (2012). Potential of chitosan-loaded nanoemulsions to control different *Colletotrichum* spp. and maintain quality of tropical fruits during cold storage. J. Appl. Microbiol..

[108] Ohashi T.L., Pilon L., Spricigo P.C., Miranda M., Correa D.S., Ferreira M.D. (2015). Post-harvest quality of ‘Golden’ papayas (*Carica papaya* L.) coated with carnauba wax nanoemulsions. Rev. Iber. Tecnología Postcosecha..

[109] Zahid N., Alderson1 P.G., Ali A., Maqbool M., Manickam S. (2013). in vitro Control of *Colletotrichum gloeosporioides* by Using Chitosan Loaded Nanoemulsions. Acta Hortic..

[110] Deng Z., Jung J., Simonsen J., Wang Y., Zhao Y. (2017). Cellulose Nanocrystal Reinforced Chitosan Coatings for Improving the Storability of Post-harvest Pears Under Both Ambient and Cold Storages. J. Food Sci..

[111] Shi S., Wanga W., Liu L., Wu S., Wei Y., Li W. (2013). Effect of chitosan/nano-silica coating on the physicochemical characteristics of longan fruit under ambient temperature. J. Food Eng..

[112] Song H., Yuan W., Jin P., Wang W., Wang X., Yanga L., Zhang Y. (2016). Effects of chitosan/nano-silica on post-harvest quality and antioxidant capacity of loquat fruit during cold storage. Post-Harvest Biol. Technol..

[113] Yu Y., Zhang S., Ren Y., Li H., Zhang X., Di J. (2012). Jujube preservation using chitosan film with nano-silicon dioxide. J. Food Eng..

[114] Davarpanaha S., Tehranifara A., Davarynejada G., Abadíab J., Khorasani R. (2016). Effects of foliar applications of zinc and boron nano-fertilizers on pomegranate (*Punica granatum cv. Ardestani*) fruit yield and quality. Sci. Hortic..

